# Users’ Experiences With the NoHoW Web-Based Toolkit With Weight and Activity Tracking in Weight Loss Maintenance: Long-term Randomized Controlled Trial

**DOI:** 10.2196/29302

**Published:** 2022-01-10

**Authors:** Elina Mattila, Susanne Hansen, Lise Bundgaard, Lauren Ramsey, Alice Dunning, Marlene N Silva, Marja Harjumaa, Miikka Ermes, Marta M Marques, Marcela Matos, Sofus C Larsen, Jorge Encantado, Inês Santos, Graham Horgan, Ruairi O'Driscoll, Jake Turicchi, Cristiana Duarte, António L Palmeira, R James Stubbs, Berit Lilienthal Heitmann, Liisa Lähteenmäki

**Affiliations:** 1 VTT Technical Research Centre of Finland Ltd Espoo Finland; 2 MAPP Centre Aarhus University Aarhus Denmark; 3 School of Psychology Faculty of Medicine and Health University of Leeds Leeds United Kingdom; 4 Centro de Investigação em Desporto, Educação Física, Exercício e Saúde Faculdade de Educação Física e Desporto Universidade Lusófona de Humanidades e Tecnologias Lisbon Portugal; 5 Comprehensive Health Research Centre NOVA Medical School Universidade Nova de Lisboa Lisbon Portugal; 6 Center for Research in Neuropsychology and Cognitive Behavioral Intervention University of Coimbra Coimbra Portugal; 7 Research Unit for Dietary Studies The Parker Institute Bispebjerg and Frederiksberg Hospital The Capital Region Denmark; 8 Centro Interdisciplinar De Estudo Da Performance Humana Faculdade de Motricidade Humana Universidade de Lisboa Lisbon Portugal; 9 Applied Psychology Research Center Capabilities & Inclusion ISPA – Instituto Universitário Lisbon Portugal; 10 Laboratório de Nutrição Faculdade de Medicina Universidade de Lisboa Lisbon Portugal; 11 Biomathematics and Statistics Scotland Aberdeen United Kingdom; 12 Department of Public Health Section for General Practice University of Copenhagen Copenhagen Denmark

**Keywords:** digital behavior change intervention, user experience, technology acceptance, weight-loss maintenance, focus groups, mixed methods, mobile phone

## Abstract

**Background:**

Digital behavior change interventions (DBCIs) offer a promising channel for providing health promotion services. However, user experience largely determines whether they are used, which is a precondition for effectiveness.

**Objective:**

The primary aim of this study is to evaluate user experiences with the NoHoW Toolkit (TK)—a DBCI that targets weight loss maintenance—over a 12-month period by using a mixed methods approach and to identify the main strengths and weaknesses of the TK and the external factors affecting its adoption. The secondary aim is to objectively describe the measured use of the TK and its association with user experience.

**Methods:**

An 18-month, 2×2 factorial randomized controlled trial was conducted. The trial included 3 intervention arms receiving an 18-week active intervention and a control arm. The user experience of the TK was assessed quantitatively through electronic questionnaires after 1, 3, 6, and 12 months of use. The questionnaires also included open-ended items that were thematically analyzed. Focus group interviews were conducted after 6 months of use and thematically analyzed to gain deeper insight into the user experience. Log files of the TK were used to evaluate the number of visits to the TK, the total duration of time spent in the TK, and information on intervention completion.

**Results:**

The usability level of the TK was rated as satisfactory. User acceptance was rated as modest; this declined during the trial in all the arms, as did the objectively measured use of the TK. The most appreciated features were weekly emails, graphs, goal setting, and interactive exercises. The following 4 themes were identified in the qualitative data: engagement with features, decline in use, external factors affecting user experience, and suggestions for improvements.

**Conclusions:**

The long-term user experience of the TK highlighted the need to optimize the technical functioning, appearance, and content of the DBCI before and during the trial, similar to how a commercial app would be optimized. In a trial setting, the users should be made aware of how to use the intervention and what its requirements are, especially when there is more intensive intervention content.

**Trial Registration:**

ISRCTN Registry ISRCTN88405328; https://www.isrctn.com/ISRCTN88405328

**International Registered Report Identifier (IRRID):**

RR2-10.1136/bmjopen-2019-029425

## Introduction

### Background

Digital behavior change interventions (DBCIs) have the potential to enable scalable solutions for health promotion and disease prevention, and some of them have been found to be effective in weight management [1,2]. The acceptance and use of DBCIs are influenced by several factors. The quality and usability of the DBCI, including the quality and trustworthiness of information and interaction, trust in the privacy and security of the DBCI, and the ease of adoption and use are important for engaging with an intervention [3]. Specific features associated with more active use include frequent updates such as new information being uploaded or new lessons becoming available, and dialogue support features such as reminders and suggestions [4,5]. The inclusion of interaction with a counselor and social interaction have also been found to increase engagement [3-5]. In addition, user- and setting-related factors have an impact on the use of DBCIs, for example, the motivation and agency of the user, the user’s personal life situation, and the study setting and recruitment strategies [3,4]. Participants have been found to commit to DBCIs more strongly in randomized controlled trials (RCTs) than in pilot studies and real-life observational studies [4].

The use of a DBCI is required for the users to become exposed to its behavior change mechanisms and gain benefits. The amount and manner of use required to gain benefits may differ between different types of interventions and also between individuals [6,7]. Therefore, it is important to track how users interact with the intervention and their experiences with it to empirically establish and define effective engagement for a new intervention [6].

Technology acceptance models aim to explain and predict user acceptance and adoption of technologies [8]. One of the most widely used models is the Technology Acceptance Model (TAM), originally developed for technologies used in organizational settings [9]. The TAM model consists of 2 main factors that influence whether users will adopt a technology, namely *perceived usefulness*, which is defined as the users’ belief that using the system will enhance their job performance, and *perceived ease of use*, which is defined as the users’ belief that using the system will be effortless. Different variations and extensions of the TAM model have been developed, including the TAM for Mobile Services (TAMM) [10]. The TAMM adds the dimensions of *perceived ease of adoption* and *trust* and extends the concept of perceived usefulness to *perceived value*. The TAMM model has been previously used for the development and evaluation of DBCIs [11,12].

Traditionally, user acceptance and experiences have been studied in the intervention development phase primarily using qualitative methods. This is an essential part of intervention development to capture the needs and perspectives of the end users [13]. User experiences and the relationship between the user and the technology change over time and in different contexts [14,15]. If a technology is intended for long-term use, the long-term experience determines whether the users will continue to use and recommend the technology to others [15]. Therefore, user experiences should be measured repeatedly during long-term use and in situations where the users are independently using the technologies.

Although quantitative measures of user experience indicate the perceived usability and value, qualitative methods are required to gain a deeper understanding of the reasons associated with user experiences. Focus groups can be used to gather such data from groups of individuals in specific situations. These investigations gather detailed user perspectives on satisfaction or dissatisfaction with services or products that are not obtained by quantitative approaches [16].

### Objectives

The NoHoW Toolkit (TK) is a DBCI for WLM (weight loss maintenance) comprising modular intervention content and integrating data from self-assessments, activity trackers, and weight scales. The content of the TK was built on evidence-based theories and techniques of behavior change targeting different psychosocial constructs, which were translated into a digital format [17]. This study employed a mixed methods approach to investigate user experiences during 12 months of TK use. The objectives of this study are as follows: (1) to quantitatively analyze the user experiences of the TK and the changes in user experiences over 1 year; (2) to investigate the use of the TK and its associations with user experience and weight outcomes; and (3) to qualitatively analyze the main strengths, weaknesses, and improvement needs of the TK and the external factors affecting user acceptance of the TK.

## Methods

### Study Procedures and Materials

#### Overview

The NoHoW trial was an 18-month, 3-center, 2×2 factorial, single-blind RCT that evaluated the efficacy of the TK in WLM. In total, 1627 participants were recruited from 3 countries—the United Kingdom, Denmark, and Portugal and randomly assigned to 4 arms—(1) control arm (400/1627, 24.58%), (2) *motivation and self-regulation* arm (403/1627, 24.76%), (3) *emotion regulation* arm (416/1627, 25.56%), and (4) *combined* arm (408/1627, 25.07%). The participants were required to be aged ≥18 years, have a verified ≥5% weight loss in the last 12 months with current weight at least 5% below their highest weight, and have had a BMI of ≥25 kg/m^2^ before weight loss. Recruitment was conducted through several channels to reach eligible individuals, for example through commercial and municipal weight loss services, registered dieticians and nutritionists, leisure centers, and local and national media coverage and advertisements. The individuals were directed to country-specific recruitment websites and completed a web-based eligibility screener. Eligible individuals were contacted for a telephonic screening interview and provided with study information. Eligible participants were invited to a clinical investigation day where informed consent was obtained before randomization. A detailed description of the study is presented in the paper by Scott et al [18].

#### Intervention

All the participants received commercial wireless body weight scales (Fitbit Aria [Fitbit LLC]), activity trackers (Fitbit Charge 2 [Fitbit LLC]), and access to the TK website with content tailored to their respective arm. The users also had access to the Fitbit smartphone app (Fitbit LLC) and although they were asked not to use it, access could not be prevented. The TK contained a dashboard and graphs for summarizing the measurements and visualizing long-term progress and enabled simple self-assessments of mood and satisfaction with diet, sleep, activity, and weight as star ratings (1 to 5 stars), and entering free text personal notes into a diary. These parts of the TK were also available to the control arm along with the self-tracking devices. The participants in the 3 intervention arms received intervention content in the form of weekly sessions displayed in the TK as an interactive map. The *motivation and self-regulation* arm had 17 sessions with an estimated minimum time duration of 51.2 minutes (ranging from 20 seconds to 6 minutes and 16 seconds per session), the *emotion regulation* arm had 17 sessions with an estimated minimum time duration of 83.3 minutes (ranging from 40 seconds to 21 minutes and 52 seconds per session), and the *combined* arm had 34 sessions combining the contents of the other 2 intervention arms and having an estimated minimum time duration of 118.3 minutes (ranging from 20 seconds to 12 minutes and 32 seconds per session). Intervention participants were encouraged to complete the intervention sessions during the first 18 weeks of the trial. This was achieved by sending participants weekly emails during this time to introduce the weekly themes and remind them to visit the TK. The control arm also received weekly emails for the first 18 weeks, but they only contained links to generic weight management content. The TK was designed to provide automatic individualized feedback to the *motivation and self-regulation* and *combined* arms based on weight, activity, sleep, and use data. However, owing to an error, the *emotion regulation* arm received the messages instead of the *motivation and self-regulation* arm. The feedback was displayed in the TK as short statements (eg, “your weight management appears better when you are more active”). The TK also provided extra support for weight regain situations (*weight alert*), where an extra module was launched if the user was >3% above their target weight. The TK implementation was fixed for the duration of the trial, that is, no new features or content were added. Only technical errors were rectified when they were reported by the trial staff. A detailed description of the TK is presented by Marques et al [17].

#### Questionnaires

Quantitative user experience data were collected through electronic questionnaires at 1 (user experience questionnaire at month 1 [UX1]), 3 (user experience questionnaire at month 3 [UX3]), 6 (user experience questionnaire at month 6 [UX6]), and 12 (user experience questionnaire at month 12 [UX12]) months. Table 1 presents the measures in each questionnaire and Multimedia Appendices 1 and 2 contain the detailed questionnaires. The TAMM model was used to create a questionnaire section aimed at measuring the acceptance of the intervention. The TAMM-based items were slightly different in the UX1 from those in the other questionnaires to capture first impression experiences. The System Usability Scale (SUS) was included as a validated measure of the system usability [19]. In addition, items measuring the overall impressions of the TK and its individual features were included. The questionnaires had voluntary open-ended items for providing free-form feedback. Furthermore, the eHealth Literacy Scale (eHEALS) was included in the baseline questionnaire of the RCT to describe the participants’ baseline capacity to engage with eHealth interventions [20].

**Table 1:** Summary of user experience measures collected through questionnaires.

Measure	Time points, month	Description
eHealth Literacy Scale [20]	0 (Baseline)	eHealth Literacy Scale, consisting of 8 statements measured on a 5-point Likert scale (1=strongly disagree to 5=strongly agree). Higher scores indicate higher digital literacy.
Technology Acceptance Model for Mobile Services, first impressions	1	User acceptance section with 14 items on perceived ease of adoption (3 items), perceived ease of use (4 items), perceived value (4 items), and trust (3 items). Rated on a 5-point Likert scale (1=strongly disagree to 5=strongly agree).
Technology Acceptance Model for Mobile Services, long-term use	3, 6, 12	User acceptance section with 13 items on perceived ease of adoption (2 items), perceived ease of use (3 items), perceived value (5 items), and trust (3 items). Rated on a 5-point Likert scale (1=strongly disagree; 5=strongly agree).
Overall score	1, 3, 6, 12	“What overall score would you give to the service?” Rated on a scale from 0 to 10 (0=very bad; 10=very good).
Recommendation	1, 3, 6, 12	“How likely is it that you would recommend the service to a friend or colleague?” Rated on a scale from 0 to 10 (0=not at all likely; 10=extremely likely).
Intention to continue using the TK^a^	1, 3, 6, 12	“How likely is it that you would consider using the service in the future?” Rated on a scale from 0 to 10 (0=not at all likely; 10=extremely likely).
System Usability Scale [19]	3, 6, 12	System usability measured with 10 items assessed on 5-point Likert scale.
TK feature ratings	3, 6, 12	The 14 main TK features were rated for their importance, ease of use, convenience, enjoyability, satisfaction, and motivation to continue using them on 5-point Likert scales. For the control arm, this section only contained the 5 TK features available to them.
Feedback on the TK	1, 3, 6, 12	Open-ended item: “If you have any other feedback on the TK, you may write it here.”
Self-assessment of use	3, 6, 12	“On average, how frequently did you use the TK during the study” and “What option describes your TK use behavior best?” Options are (1) “I used the TK very constantly during the whole study,” (2) “I used the TK more in the beginning of the study,” (3) “I used the TK more in the end of the study,” (4) “I quit using the TK in the middle of the study,” and (5) “Other.”
Email	3, 6, 12	“I like receiving email from the TK.” Rated on a 5-point Likert scale (1=strongly disagree; 5=strongly agree)
Open-ended questions	3, 6, 12	Open-ended items: (1) “What motivated you to continue using the TK,” (2) “Why did you use the TK more in the beginning than in the end of the study,” and (3) “Why did you quit using the TK in the middle of the study?”

^a^TK: Toolkit.

#### Focus Group Discussions

Focus group discussions were conducted after 6 months. Focus groups were organized to provide a deeper understanding of the user experiences of the TK. A participant was eligible if they had been using the TK for ≥6 months. In each country, 1 focus group per arm was conducted, leading to a total of 12 groups. Recruitment was done by listing all the participants who had the opportunity to use the TK for ≥6 months at the time of the focus group discussions and recruiting from this list until 8 participants agreed to participate. Participants were recruited via an email that included an invitation letter and an information sheet. Groups were moderated by one or more researchers who were experienced in conducting focus groups and qualitative research and who had not taken part in the TK design or development or participated in delivering the intervention (LB [Denmark], MNS [Portugal], and AD and LR [United Kingdom]). The conversations were guided by a semistructured discussion guide, which highlighted the following 5 main topics of conversation: ways of use, user experience, perceived support in WLM, impact on weight management, and how to improve the TK (Multimedia Appendix 3). Unplanned topics of conversation were also explored based on the issues raised by the participants. Moderators aimed to ensure that no one participant dominated the conversation, and every participant was given the opportunity to contribute to discussions. Ethical approval was granted by the local institutional ethics committees at the Universities of Leeds (17-0082; February 27, 2017), Lisbon (17/2016; February 20, 2017) and the Capital Region of Denmark (H-16030495; March 8, 2017).

### Analysis

#### Quantitative Data Analysis

The TK use was captured in log files, which were used to calculate the total number of visits to the TK and the total duration of use during the 12 months of the study. The percentages of weekly users and retained users were plotted for the control and intervention arms. Retention was determined based on rolling retention, which means that a participant was considered as a retained user if they used the TK during or after a specific week. Intervention completion was calculated for the intervention arm participants based on the duration of time they spent in the sessions assigned to them versus the estimated duration of the sessions. For most sessions, if a user visited a session at least once and remained engaged for at least 33% of the estimated duration, the session was considered as completed. For sessions containing video and audio content, the threshold was increased, that is, the user was required to spend 50%-80% of the estimated duration in the session. The completion rates were calculated as the percentage of completed sessions compared with the total number of sessions in the arm. The use metrics were reported as medians and IQRs owing to the skewed distributions and nonparametric methods were used for comparisons and correlations (ie, Kruskal-Wallis test, Mann-Whitney *U* test, and Spearman correlation). Spearman correlations between use metrics and weight changes from baseline to 12 months were calculated for each intervention group.

The statistics of responses to the questionnaires were summarized. Quantitative questionnaire sections were descriptively summarized using means and SDs. One-way analysis of variance and Tukey post hoc test were used to determine if there were differences between the intervention arms, and Spearman correlations between the number of visits and the user experience items were calculated. The TAMM-based items were summarized as mean scores over the 4 TAMM dimensions and a total score over all the items. The eHEALS score was calculated as the sum of individual items. The SUS was scored according its guidelines [19]. Principal component analysis was conducted on the 6 items that measured the appeal of the TK features to investigate whether there were underlying or latent variables that accounted for the items. Differences between the centers were investigated by comparing use metrics, eHEALS score, and 3-month user experiences in the control and intervention arms using a general linear model. For this analysis, a logarithmic transformation was applied to the number of visits and total duration of use to normalize their distribution. Quantitative data were analyzed using MATLAB R2017a (Mathworks) and IBM SPSS Statistics version 26 (IBM Corporation). An α level of .05 was used as the threshold for statistical significance.

#### Qualitative Data Analysis

Focus groups were transcribed and analyzed thematically via an iterative, nonlinear, and nonprescriptive process [21,22]. This involved initial familiarization with the transcripts, reflections on similarities and differences between cases, and systematic coding of the data. After preliminary discussions across the countries on codes and themes, the initial coding framework was developed in Denmark and further iterated collaboratively to integrate the findings from Portugal and the United Kingdom. In each country, experienced researchers were involved in the process and coding was done from original transcripts by native speakers (LB and LL [Denmark], MNS [Portugal], and AD and LR [United Kingdom]). At each stage, decisions on coding and analysis were discussed and revised by all coders and where necessary, the original data sources were revisited to ensure that the decisions were grounded in the data (refer the focus group code book in Multimedia Appendix 4). The analysis was particularly focused on the research questions (ie, the strengths, weaknesses, and improvement needs), while considering unpredicted but relevant themes. Similarly, responses to the open-ended questionnaire items were also thematically analyzed separately but by using the code book of the focus group analysis as the starting point. The analyses were conducted using NVivo (version 12; QSR International).

## Results

### Response to Questionnaires

On average, the participants responded to the UX1 after 33.7 (SD 33.3) days, to the UX3 after 94.8 (SD 25.6) days, to the UX6 after 196 (SD 22.3) days, and to the UX12 after 378 (SD 31.2) days from the first log in to the TK, which indicated that the timing of the questionnaires was realized according to the plan.

Response rates varied between the questionnaires, with the highest response rate in the UX1 (1096/1627, 67.36%) and the lowest in the UX6 (829/1627, 50.95%). Of the 1627 participants, 1383 (85%) responded to at least 1 of the questionnaires and 427 (26.24%) responded to all the 4 questionnaires, with an approximately even distribution among the arms (110/427, 25.7% respondents in the control arm, 102/427, 23.9% in the *motivation and self-regulation* arm, 114/427, 26.7% in the *emotion regulation* arm, and 101/427, 23.7% in the *combined* arm). The focus groups reached their recruitment target and involved 4.55% (74/1627) participants in total, with a mean of 6.2 (SD 1.3) participants per discussion.

Of the total study sample, 68.65% (1117/1627) were women. The proportion of female participants was higher in the United Kingdom and Denmark (442/555, 79.6% and 429/536, 80%, respectively) than in Portugal (246/536, 45.9%). The mean age of participants was 44.1 (SD 11.9) years, with the oldest participants in Denmark with a mean age of 47.6 (SD 11.5) years, followed by the United Kingdom with a mean age of 44.4 (SD 12.9) years and Portugal with a mean age of 40.1 (SD 9.7) years. The mean BMI was 29.7 (SD 5.3) kg/m^2^ at baseline, 30.3 (SD 5.7) kg/m^2^ in the United Kingdom, 30.7 (SD 5.3) kg/m^2^ in Denmark, and 28.0 (SD 4.5) kg/m^2^ in Portugal.

In the UX1, 33.57% (368/1096) of participants responded to the open-ended question. In total, 92.9% (897/966) of participants responded to at least 1 open-ended question in the UX3, 94.2% (781/829) in the UX6, and 97.3% (871/895) in the UX12.

### Use Activity

In total, 98.59% (1604/1627) participants logged into the TK at least once. Table 2 summarizes the use metrics in the arms. According to the Kruskal-Wallis test, the total duration of use and completion differed significantly between the arms (*P*<.001 for both). Post hoc tests showed that the total duration of use in the control arm was significantly lower than that in the other arms (Mann-Whitney *U* test *P*<.001 for all pairwise comparisons) and that the *combined* arm had a significantly longer duration of use and lower completion percentage than the other intervention arms (Mann-Whitney *U* test *P*<.001 for both pairwise comparisons).

**Figure 1:**
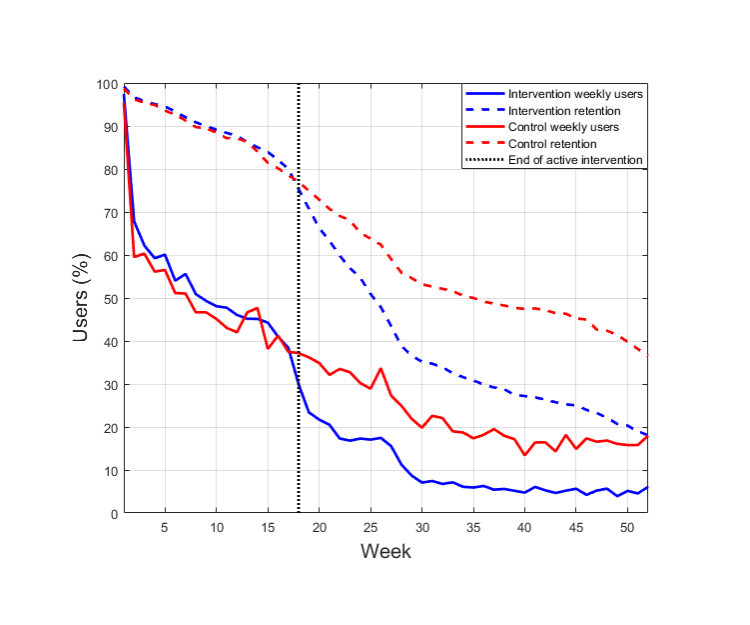
Percentage of actual weekly users and retained users according to the rolling retention criterion by study week in the intervention arms and control arm.

Figure 1 shows the percentage of users in the control and intervention arms (combined), accessing the TK during each week of the study and the rolling retention for the same period. The figure shows that although the number of actual weekly users declined rapidly at the beginning, 75.35% (1226/1627) of the users were retained until week 18, that is, the end of the active intervention.

In all the questionnaires, the respondents used the TK significantly more than the nonrespondents (*P*<.001 for all use metrics in all the questionnaires; data not shown).

The correlations between the TK use and weight outcomes were small, but showed that a higher amount of TK use was associated with weight loss (Table 3). The correlations were strongest in the *combined* arm, followed by the control arm.

**Table 2:** Use metrics by arm (N=1627).

Arm	Participant, n (%)	Number of visits, median (IQR)	Total duration of use (minutes), median (IQR)	Completion percentage, median (IQR)
Control	400 (24.59)	16 (7-36)	54.5 (21.2-124.1)	N/A^a^
Motivation and self-regulation	403 (24.77)	16 (9-25.75)	146 (77.4-238.4)	76.2 (47.6-90.5)
Emotion regulation	416 (25.57)	15 (9-24)	132.1 (74.3-237.5)	73.7 (47.4-89.5)
Combined	408 (25.08)	17 (10-27.5)	215.4 (112.3-333.2)	64.9 (5.1-83.8)

^a^N/A: not applicable.

**Table 3:** Spearman correlations between weight change from baseline to 12 months and use metrics.

Arm	Visits			Total duration			Completion	
	*ρ* ^a^	*P* value	*ρ*		*P* value	*ρ*		*P* value
Control	–0.125	.03	–0.114		.04	N/A^b^		N/A
Motivation and self-regulation	–0.083	.14	–0.072		.21	–0.132		.02
Emotion regulation	–0.092	.10	–0.136		.02	–0.106		.06
Combined	–0.198	<.001	–0.175		.002	–0.167		.003

^a^Spearman correlation.

^b^N/A: not applicable.

### Quantitative User Experience Results

#### Digital Literacy

The average eHealth literacy score measured with the eHEALS questionnaire at baseline was 30.8 (SD 5.12; range 10-40) out of a maximum of 40, with higher values signifying higher levels of eHealth literacy. There were no significant differences between the arms and no significant correlations between digital literacy levels and use (data not shown); thus, eHealth literacy is not expected to have affected the results.

#### Usability

The average SUS scores for measuring the usability of the TK decreased over time in the control arm, from a mean of 70.4 (SD 21.3) in the UX3 to 67.6 (SD 19.9) in the UX12; in the *motivation and self-regulation* arm, from 68.4 (SD 20.9) in the UX3 to 62.1 (SD 20.2) in the UX12; in the *emotion regulation* arm, from 73.0 (SD 19.2] in the UX3 to 67.1 (SD 19.8) in the UX12; and in the *combined* arm, from 69.9 (SD 20.2) in the UX3 to 66.2 (SD 19.5) the UX12. The lowest scores were obtained in the *motivation and self-regulation* arm and they differed significantly from those in the *emotion regulation* arm (*P*=.01) in the UX3 and from all other arms in the UX12 (control arm: *P*=.003; *emotion regulation* arm: *P*=.007; *combined* arm: *P*=.03).

In the control arm, the SUS scores correlated significantly with the number of visits to the TK in all the questionnaires (*ρ*=0.426 in the UX3, *ρ*=0.362 in the UX6, and *ρ*=0.434 in the UX12; all *P*<.001). In the *motivation and self-regulation* arm, there was a small but significant correlation only in the UX3 (*ρ*=0.142; *P*=.03). In the *emotion regulation* arm, there were significant correlations in all the questionnaires (*ρ*=0.186, *P*=.003 in the UX3; *ρ*=0.164, *P*=.02 in the UX6; and *ρ*=0.218, *P*=.001 in the UX12). In the *combined* arm, there were no significant correlations between SUS scores and number of visits.

#### Overall Score, Recommendation, and Intention to Continue Using the TK

In most questionnaires, the intervention arm participants gave the TK significantly higher ratings in overall score, likelihood of recommending, and intention to continue to use the TK compared with the control arm participants; ratings declined in all the arms during the study. The ratings mostly did not differ among the 3 intervention arms; only in the UX6, the *combined* arm participants gave a higher overall score than the other intervention arms and had a higher intention to continue than the *emotion regulation* arm (*P*=.01). There were small but significant correlations between the ratings and the number of visits to the TK in the control and *emotion regulation* arms (Table 4).

**Table 4:** Overall score and ratings for the likelihood of recommending and intention to continue and their correlation with the number of visits.

UX^a^ and arm			Overall score					Likelihood of recommending					Intention to continue		
			Score, mean (SD)		*ρ* ^b^		Score, mean (SD)			*ρ*		Score, mean (SD)			*ρ*
**UX at month 1**															
	Control	6.74 (2.19)		0.117^c^		6.18 (3.01)			0.125^c^		6.31 (2.82)			0.120^c^	
	Motivation and self-regulation	7 (2.05)		0.036		7.08 (2.64)			–0.053		6.97 (2.66)			0.034	
	Emotion regulation	7.3 (1.85)		0.175		7.04 (2.75)			0.134^c^		7.13 (2.53)			0.121^c^	
	Combined	7.23 (1.86)		0.037		7.15 (2.58)			0.060		7.26 (2.36)			0.079	
**UX at month 3**															
	Control	6.21 (2.41)		0.253^d^		5.92 (3.19)			0.143^c^		5.88 (3.04)			0.243^d^	
	Motivation and self-regulation	6.56 (2.24)		0.114		6.75 (2.87)			0.049		6.67 (2.7)			0.113	
	Emotion regulation	6.38 (2.34)		0.189^d^		6.46 (2.95)			0.169^d^		6.31 (3.01)			0.183^d^	
	Combined	6.75 (2.09)		0.003		6.83 (2.78)			–0.015		6.68 (2.8)			0.026	
**UX at month 6**															
	Control	5.95 (2.47)		0.262^d^		5.58 (3.27)			0.281^d^		5.39 (3.22)			0.325^d^	
	Motivation and self-regulation	6.16 (2.22)		0.036		6.22 (3)			0.026		5.84 (2.88)			0.055	
	Emotion regulation	6.04 (2.44)		0.163^c^		6.01 (3.24)			0.157^c^		5.56 (3.15)			0.210^d^	
	Combined	6.66 (2.18)		0.103		6.63 (2.89)			0.059		6.32 (2.86)			0.129	
**UX at month 12**															
	Control	5.39 (2.46)		0.279^d^		4.62 (3.28)			0.241^d^		4.35 (3.33)			0.263^d^	
	Motivation and self-regulation	5.9 (2.33)		0.047		5.77 (3.06)			0.010		5.07 (3.12)			0.060	
	Emotion regulation	5.85 (2.48)		0.169^c^		5.36 (3.37)			0.201^d^		4.8 (3.28)			0.201^d^	
	Combined	6.1 (2.4)		0.002		5.81 (3.17)			0.020		5.38 (3.12)			0.061	

^a^UX: user experience questionnaire.

^b^Spearman correlation between user experience ratings and number of visits to the Toolkit.

^c^Correlation is significant at the significance level of *P*<.05.

^d^Correlation is significant at the significance level of *P*<.01.

#### TAMM Model

Table 5 presents the mean scores for all the items and the 4 TAMM dimensions. For the total score, the results are presented for all the arms separately, whereas in other results, the intervention arms are combined, as the total score mostly did not differ among the intervention arms. In the UX1, the post hoc tests showed that the *emotion regulation* arm participants gave a higher total score to the TK than the *combined* arm participants (*P*=.006) and the control arm participants (*P*=.02).

The participants of the intervention arms gave a higher rating for *value* in all the questionnaires (*P*<.001 in the UX1, UX6, and UX12 and *P*=.01 in the UX3). The ratings for *ease of adoption*, *ease of use*, and *trust* were mostly similar in the control and intervention arms in all the questionnaires; only in the UX6, the intervention arms had a higher score for *ease of use* (*P*=.02) and in the UX3, for *trust* (*P*=.04). All the ratings decreased during the trial, and only the ratings for *trust* remained above 4 in the intervention arms. In both the control and intervention arms, the highest scores were given to *trust* and the lowest scores to *value*.

When analyzing only the participants who responded to all the user experience questionnaires, the questionnaire averages and the decline during the trial were nearly identical, that is, the decline was not because of the differences in the respondent populations (data not shown).

Table 6 presents correlations between the total number of visits and TAMM total score in all the arms. The control arm showed the highest correlations between the TAMM score and TK use in all the questionnaires.

**Table 5:** Means and SD of Technology Acceptance Model for Mobile Services (TAMM) scores.

Characteristic and arm		UX1^a^	UX3^b^	UX6^c^	UX12^d^
**TAMM total score, mean (SD)**					
	Control arm	3.83 (0.71)	3.49 (0.76)	3.37 (0.77)	3.22 (0.77)
	Motivation and self-regulation	3.80 (0.75)	3.55 (0.73)	3.52 (0.68)	3.36 (0.75)
	Emotion regulation	3.97 (0.65)	3.64 (0.70)	3.51 (0.76)	3.37 (0.79)
	Combined	3.9 (0.71)	3.6 (0.74)	3.51 (0.74)	3.38 (0.77)
**Ease of adoption, mean (SD)**					
	Control arm	4.06 (0.86)	3.75 (0.95)	3.74 (0.93)	3.64 (1)
	Intervention arms	3.98 (0.86)	3.7 (0.85)	3.61 (0.93)	3.52 (0.96)
**Ease of use, mean (SD)**					
	Control arm	4.08 (0.86)	3.58 (0.91)	3.44 (0.92)	3.36 (0.89)
	Intervention arms	3.98 (0.87)	3.66 (0.84)	3.6 (0.84)	3.49 (0.88)
**Value^e^, mean (SD)**					
	Control arm	3.18 (1)	2.99 (1)	2.77 (1.04)	2.56 (0.98)
	Intervention arms	3.5 (0.92)	3.17 (0.93)	3.05 (0.95)	2.86 (0.98)
**Trust, mean (SD)**					
	Control arm	4.13 (0.75)	4.08 (0.77)	4.04 (0.80)	3.91 (0.89)
	Intervention arms	4.20 (0.71)	4.19 (0.69)	4.14 (0.71)	4.01 (0.77)

^a^UX1: user experience questionnaires at month 1.

^b^UX3: user experience questionnaires at month 3.

^c^UX6: user experience questionnaires at month 6.

^d^UX12: user experience questionnaires at month 12.

^e^Value is one of the TAMM model dimensions.

**Table 6:** Spearman correlations between the Technology Acceptance Model for Mobile Services total score and number of visits to the Toolkit.

Arm	UX1^a^, *ρ*	UX3^b^, *ρ*	UX6^c^, *ρ*	UX12^d^, *ρ*
Control	0.193^e^	0.409^e^	0.342^e^	0.379^e^
Motivation and self-regulation	0.142^f^	0.199^e^	0.036	0.059
Emotion regulation	0.126^f^	0.168^e^	0.187^e^	0.247^e^
Combined	0.139^f^	0.065	0.141^f^	0.1

^a^UX1: user experience questionnaires after month 1.

^b^UX3: user experience questionnaires after month 3.

^c^UX6: user experience questionnaires after month 6.

^d^UX12: user experience questionnaires after month 12.

^e^Correlation is significant at the significance level of *P*<.01.

^f^Correlation is significant at the significance level *P*<.05.

#### Feature Ratings

Principal component analysis was conducted on the 6 appeal items rated on each feature. In all the questionnaires, only 1 factor was found, accounting for 65.2% to 68.9% of the variance in the items in the UX12 and UX3, respectively. The component loadings were similar for all the items, and therefore, the mean of the appeal items was calculated to represent the perception of the features (Table 7).

All the feature ratings were between 3 and 4 on a 5-point scale. At 3 months, the intervention arm participants gave the highest ratings to weekly emails, graphs, and goal setting; at 6 months to graphs, weekly emails, and interactive exercises; and at 12 months, they gave the highest ratings to graphs, interactive exercises, and goal setting. The control arm participants gave the highest ratings to weekly emails and graphs at 3 and 6 months and to graphs and dashboard at 12 months.

**Table 7:** Ratings for the main features of the Toolkit. Control arm scores are shown only for the features available for them.

Feature and arm			UX3^a^, mean (SD)		UX6^b^, mean (SD)		UX12^c^, mean (SD)
Interactive map			3.52 (1.06)		3.42 (1.03)		3.28 (1.03)
Theme introduction videos			3.67 (1)		3.55 (0.98)		3.41 (1.01)
Text information			3.75 (0.92)		3.66 (0.93)		3.47 (0.91)
Interactive exercises			3.78 (0.96)		3.7 (0.97)		3.55 (0.99)
Audio exercises			3.45 (1.13)		3.4 (1.08)		3.31 (1.1)
**Dashboard**							
	Control arm	3.79 (0.9)		3.64 (0.88)		3.45 (0.95)	
	Intervention arms	3.78 (0.94)		3.65 (0.95)		3.5 (0.97)	
**Graphs**							
	Control arm	3.82 (0.91)		3.72 (0.93)		3.55 (0.96)	
	Intervention arms	3.88 (0.97)		3.77 (0.96)		3.66 (0.97)	
Goal setting			3.85 (0.93)		3.66 (0.97)		3.52 (1.00)
Coping and action plan			3.65 (0.97)		3.51 (0.98)		3.35 (1.03)
**Personal notes**							
	Control arm	3.38 (1.05)		3.17 (1.07)		3.07 (1.12)	
	Intervention arms	3.28 (1.05)		3.26 (1.02)		3.16 (1.03)	
Personal feedback tile			3.42 (1.04)		3.33 (1.02)		3.19 (1.06)
Weight alert			3.78 (1.07)		3.67 (1.06)		3.49 (1.14)
**Summary tile**							
	Control arm	3.57 (1.02)		3.45 (0.91)		3.31 (1.04)	
	Intervention arms	3.6 (1.03)		3.49 (1.01)		3.36 (1.04)	
**Weekly emails**							
	Control arm	3.83 (0.97)		3.68 (1.02)		3.19 (1.16)	
	Intervention arms	3.94 (0.95)		3.71 (1.02)		3.42 (1.12)	

^a^UX3: user experience questionnaires after month 3.

^b^UX6: user experience questionnaires after month 6.

^c^UX12: user experience questionnaires after month 12.

#### Center Differences

Age and gender were added to the general linear models as covariates, as these background variables differed between the centers. There were no significant differences between the use metrics among the centers (Table 8).

There were differences in the eHEALS scores. The Portuguese intervention participants gave significantly lower scores than the participants in other countries. In addition, the SUS score differed among the centers in the intervention arms, and all other investigated user experience ratings differed among the centers in both the control and intervention arms. In all the user experience ratings, the highest scores were given by Portuguese participants and lowest by the Danish participants.

**Table 8:** Differences among countries in the control and intervention arms. *P* values are obtained from the general linear model with the adjustment for age and gender.

Characteristic and arm		United Kingdom	Denmark	Portugal	*P* value
**Number of visits, median (IQR)**					
	Control arm	17 (8-34)	20 (8-36)	13 (6.5-44)	.91
	Intervention arms	17 (10-26)	17 (9.5-25.5)	14 (7-24)	.12
**Total duration in minutes, median (IQR)**					
	Control arm	54.8 (23.8-140.8)	65.7 (22.8-129.6)	45.7 (13.9-105.5)	.56
	Intervention arms	158.7 (81.1-274.3)	169.8 (87.7-280.9)	140.2 (73.6-249.2)	.58
**Completion percentage of sessions, median (IQR)**					
	Control arm	N/A^a^	N/A	N/A	N/A
	Intervention arms	71.4 (46-89.5)	76.2 (47.4-89.5)	68.4 (37.8-85.7)	.78
**eHEALS^b^(range 10-40), mean (SD)**					
	Control arm	31 (5.1)	31.7 (5.34)	30.1 (4.76)	.27
	Intervention arms	31.2 (5.71)	31 (4.8)	29.8 (4.71)	.009
**SUS^c^ in UX3^d^, mean (SD)**					
	Control arm	70.8 (20.6)	65.8 (20.6)	74.6 (19.6)	.07
	Intervention arms	69.3 (20.6)	64.4 (20.5)	77.4 (17.3)	<.001
**Overall score in UX3, mean (SD)**					
	Control arm	6 (2.4)	5.38 (2.57)	7.15 (1.92)	<.001
	Intervention arms	6.66 (2.12)	5.72 (2.41)	7.38 (1.78)	<.001
**Likelihood of recommending in UX3, mean (SD)**					
	Control arm	5.39 (3.28)	4.82 (3.1)	7.39 (2.62)	<.001
	Intervention arms	6.64 (2.81)	5.69 (3.03)	7.77 (2.3)	<.001
**Intention to continue in UX3, mean (SD)**					
	Control arm	5.59 (3.17)	5.22 (3.12)	6.74 (2.67)	.001
	Intervention arms	6.63 (2.8)	5.79 (3.05)	7.30 (2.44)	<.001
**TAMM^e^ total score in UX3, mean (SD)**					
	Control arm	3.5 (0.73)	3.29 (0.79)	3.69 (0.71)	.005
	Intervention arms	3.63 (0.71)	3.34 (0.7)	3.86 (0.66)	<.001

^a^N/A: not applicable.

^b^eHEALS: eHealth Literacy Scale.

^c^SUS: System Usability Scale.

^d^UX3: user experience questionnaires at month 3.

^e^TAMM: Technology Acceptance Model for Mobile Services.

### Experiences From Focus Groups and Open-ended Responses in Questionnaires

#### Overview

Analysis of the focus groups and open-ended questionnaire data revealed the following four main themes: (1) engagement with features, (2) use decline, (3) external factors affecting user experience, and (4) suggestions for improvements. These reflect the opportunities and challenges regarding the user acceptance of a DBCI. In the following, each theme and its subthemes are described. In the qualitative data, the comments and overall perception of the TK were very similar in the 3 countries and across intervention arms; thus, the results are not divided into arms or countries. Comments from the focus groups are marked with sex, study arm, and country; he survey responses are marked with the code “UX” and the month of the survey is added to the end of the identifier.

#### Engagement With Features

During the focus groups, participants identified and discussed the features and aspects they found most helpful regarding their WLM and these were also explained in the questionnaire comments.

Support for self-monitoring was perceived positively in both the focus groups and questionnaire comments. For participants, monitoring progress in their weight, sleep, and activity through graphs, dashboards, and weight alerts was identified as very important for their continued WLM, as it gave them a sense of control over their progress. A participant from Portugal commented the following:

It is like this, it gave me a conscience, a sense of control of my body that I did not feel, I never felt this before, never....

Female, emotion regulation arm, Portugal

Although participants often highlighted the usefulness of the Fitbit app in monitoring weight, they also mentioned that the visualization of their development and progress provided by the TK through graphs and dashboards was important. In contrast, some participants did not find the monitoring as helpful and were reluctant to weigh themselves often, fearing that it would be stressful and potentially detrimental to their WLM:

I do not weigh myself every single day, because it gives the wrong impression...You become too stressed.

Female, control arm, Denmark

However, in addition to monitoring, the ability of the TK to induce self-awareness and help the participants reflect on their choices was also perceived as important. The idea that everyone must think and know what they do, regarding eating and activity, was fundamental to orient behavior in a different way:

And, my moment of reflection of the day was that, [laughter] as I looked at the weight. I thought, I have more or less weight, why? What did I do yesterday? What did I do yesterday that trigger these changes? And I usually do this reflection, while I am dressing for the day I am doing this reflection, humm, how does my body respond to what I ate yesterday?

Male, combined arm, Portugal

Participants in the focus groups perceived the TK content and intervention modules as especially useful for their WLM if they prompted them to reflect on the association between behavior and weight. The participants also acknowledged the importance of emotion regulation on WLM, regardless of whether they had it in their intervention, as illustrated by the following comments:

...It was important to be able to perceive the association between things...but I felt sad for a while,...why?’ 'Why? Do I sleep less than I need? Because am I grumpier? ' 'Because I am doing little exercise and this is also leaving me more tired, or because I am doing a silly food restriction, or because I am overeating and the food is affecting me emotionally; This, I think makes sense….

Female, motivation and self-regulation arm, Portugal

The biological side of it, about calories and exercise and those things, that’s fine. However, for me, there is also the issue of working with your habits, and the impact of emotions.

Male, motivation and self-regulation arm, Denmark

Participants further indicated that the TK allowed them to be more conscious of the behavioral patterns linked to sedentary behaviors and bad eating habits and to know themselves better.

Thus, the various features of the TK enabled participants to learn about their own behavior and better understand the antecedents and consequences of their food-related choices, thus helping them self-regulate their behavior and respond to emotional cues.

#### Use Decline

The use of the TK declined over time. The participants often linked their declining use to various design aspects that they felt hindered their use of the TK. Most often, participants expressed frustration with the login procedure:

I think it is cumbersome and illogical that you can’t change your username and password.

Male, control arm, Denmark

The focus group participants reported varying engagement with the content and sessions of the TK. Although the monitoring tools were important for progress, many of the content-heavy features failed to engage participants in the same way. A prominent issue was the time required to complete the modules as the duration often exceeded the participant’s expectations, meaning that the participants had to unwillingly take time out of their day to participate:

I think at the beginning it said they [sessions] are only about 5 minutes, you won’t have to spend very long on them. And then gradually they got longer until they were about 20 minutes when I was just sitting there listening. I’d have my earphones on but there was still sort of activity going on in the house. It didn’t say...even if it came through on the email, it didn’t say that in order to do this you need to make sure that you are giving yourself time, space and quietness.

Female, emotion regulation arm, United Kingdom

The issue was exacerbated by problems with responsiveness and readability when accessing the TK via a mobile phone, which forced the participants to access it via their home or work computers. This was not always convenient within a busy lifestyle full of interruptions and complications, which was an additional reason for declining use:

If it was an app so I could access from my phone, I would use it more often than I do now. At present I have to use it on a laptop or desktop computer which I find quite restrictive in terms of times I can use it.

UX12, male, emotion regulation arm, United Kingdom

Although some participants felt that the information provided in the TK was interesting, motivating, and relevant, some felt there was nothing new in the information or that its presentation was not pleasing:

They are sometimes a bit patronizing too. I get quite cross at being told to eat my veggies!

UX6, female, combined arm, United Kingdom

It is boring, and it doesn’t tell me anything I didn’t already know, so I stopped using it quite early, it is too dry.

UX3, female, emotion regulation arm, Denmark

In the questionnaire responses, participants across the intervention arms reported that the weekly modules were tasks that they enjoyed or needed to complete. Weekly reminders were also well received and considered very important in inviting users back to the TK. Without the reminders and the tasks to complete, motivation to use the TK declined:

Once the tasks were completed, I kind of forgot about it.

UX12, female, motivation and self-regulation arm, United Kingdom

It feels a bit like now the weekly support sessions have finished, the momentum for logging into the tool kit has gone...I enjoyed the weekly sessions, found them informative and they helped keep me 'in the zone.

UX6, female, motivation and self-regulation arm, United Kingdom

Interestingly, the control arm participants used the TK in a similar way as the intervention arms participants, despite having much less content:

First of all it was the prompting from emails from the project and now I use it as a regular part of my weight management. I like to see my progress, good or bad and when it’s bad it’s a good incentive to get back on track.

UX6, female, control arm, United Kingdom

#### External Factors Affecting the User Experience of the TK

Apart from technical issues, there were several external factors that influenced participants’ perceptions of the TK. The activity information collected via the Fitbit smartphone app was available to view on the TK, but the overall design was considered lacking, especially when compared with the Fitbit app, which was also available to all the participants alongside the activity tracker and weight scales. Furthermore, many participants were aware of other commercial weight management apps and used them as a benchmark to measure the TK. This comparison was often unfavorable, and the participants wanted future iterations of the TK to incorporate the best parts of the commercial apps or at least be compatible with them because of their convenience, accessibility, user-friendliness, and design:

I have an app on my phone that is more accessible and has a better design, and there is a long way to make the website live up to the same requirements or the same standards. If I did not have the possibility to access the Fitbit app, then I would definitely have looked more at those graphs [on the TK]. Then I would have had to go there to find the same information.

Male, motivation and self-regulation arm, Denmark

There's nothing particularly wrong with the Toolkit as such - it's just not as nice and easy to use as the Fitbit app. The Fitbit app in conjunction with [other app] provides everything I'd want, all accessible on my phone.

UX1, female, control arm, United Kingdom

Finally, study procedures such as the user experience surveys, the food recall questionnaire (INTAKE 24, which was a measurement of the NoHoW trial rather than part of the TK), and visits to the local study sites for weighing and measuring were often confused with the TK and some participants were unsure about what was meant when asked about it:

The Toolkit is very good, but the Intake diary is very frustrating.

UX1, male, emotion regulation arm, United Kingdom

When asked for feedback on the TK

The questionnaires are too long, you get annoyed answering them.

UX12, male, emotion regulation arm, Denmark

#### Improvement Needs

The focus group participants had many ideas to improve the TK. The most prevalent suggestion was the need to create an app, not only to make the service more competitive but also to circumvent issues with login and access and make it easier for the participants to prioritize and plan when to use it. The possibility of personalizing the TK according to one’s own use and interests was also highlighted. Similarly, the participants wanted the content to be more tailored to their specific needs, for example, information about the expected time allocation required for the activities and designing a tool that provides immediate help in tempting food situations:

Making it into an app would make it more aggressive, which I think in our busy lives we could all do with reminding...If you could have a more aggressive app...it would remind you and say, this is what you have asked for, this is what you want. Sometimes, personally I need reminding.

Female, emotion regulation arm, United Kingdom

...as a Toolkit user, eventually, I could have the opportunity to change the initial [Map] panel, to access all information but only having my ‘favorites’ on the initial panel...To rearrange it the way I want it.

Male, control arm, Portugal

The lack of interaction with other participants was also regarded as a weakness of the TK. Creating a social platform to allow the participants to interact with others, discuss their progress, and offer support when needed was considered important. The participants spoke about previous experiences in which social support, knowledge exchange, shared activities, and friendly competition had a positive impact on their weight loss:

I’m thinking more from a personal motivation point of view and support, while we are all undertaking the programme, wouldn’t it be great for us to interact and support each other.

Female, emotion regulation arm, United Kingdom

Furthermore, many participants asked for more feedback and notifications from the system. Although the TK provided individualized feedback in 2 arms, it was not widely discussed in the focus groups. One reason for this might be insufficient data to provide personalized feedback owing to the participants’ low level of engagement. The prompting emails sent from the TK seemed to be the main trigger for use and once the reminder messages stopped, use declined. However, the users wished they had received periodic personalized feedback emails with advice on how to reach the individual goals and believed that they would have helped them sustain their engagement:

...one monthly [email], one was enough, one monthly. Because, lately, I have not been contacted, since I came here the last [time] I did not receive any more emails, I do not know; I've already wondered, sometimes, what was I going there for, does the project still exist?

Male, motivation and self-regulation arm, Portugal

...depending on our use, of the results you are having, [hum-hum] humm, some feedback on that, i.e. you are getting these results, maybe a few suggestions of what you can do to continue towards the goals that we are defining for ourselves. This could be an important feedback.

Male, combined arm, Portugal

In addition, some participants expressed confusion as the content on the website was not updated during the trial period. Their use of other commercial services created the expectation of regular updates:

We have never seen an update; a year has passed and not one update was made...

Male, motivation and self-regulation arm, Portugal

In addition, there was some confusion among the participants regarding what data would be accessible to researchers. Although this issue was not explicitly discussed, the participants often thought that the researchers had access to all their data, which may have impacted the participants’ engagement with and use of the TK, as not receiving feedback from researchers was seen as unfair:

At the moment it seems like they must be collecting a huge amount of data, which is for the research program, but actually the people that are forming that data are not getting any of that.

Female, motivation and self-regulation arm, United Kingdom

## Discussion

### Principal Findings

We investigated the quantitative and qualitative user experiences and objectively measured the use of the TK, a digital WLM intervention, during the NoHoW RCT. Our goal was to investigate the specific factors affecting user experiences and use of the TK over a 1-year period.

TK use was high during the 18-week active intervention, when >75.35% (1226/1627) of the participants were retained, and >31.71% (516/1627) accessed the TK on a weekly basis. These rates are comparable with the high retention rates found for smartphone-based health interventions, ranging from 29% to 79.6% [23]. Later, when the users were free to use the TK at their own discretion, a rapid decline in use was seen, especially in the intervention arms. A similar effect was reported by Mattila et al [24]. For the TK, the most likely reason for the decline was the discontinuation of the email reminders and many participants felt that they had completed their tasks in the intervention. The users completed between 64.9% (*combined* arm) and 76.2% (*motivation and self-regulation* arm) of their assigned intervention sessions.

A higher amount of use was associated with 12-month weight loss in all the intervention arms with small but significant correlations. The metrics with the highest correlation differed among the arms. In the control arm, where the TK only contained self-monitoring–related features, the number of visits had the highest correlation with weight loss. In the *motivation and self-regulation* arm, which consisted of modules with textual content and interactive exercises, intervention completion had the strongest correlation with weight loss. In the *emotion regulation* arm, which consisted of significant video and audio content, the total duration of use had the highest correlation with weight loss. Finally, the *combined* arm, which combined the content of the other 2 arms, showed significant correlations for all the use metrics, with the strongest correlation being between the number of visits and weight loss. This highlights the need to investigate the TK use from different perspectives and by using different metrics. Donkin et al [7] previously reported that in physical health interventions, the number of logins was most consistently associated with effectiveness, probably because of the high emphasis on self-monitoring, whereas intervention completion was most related to effects of psychological health interventions. In this study, both types of metrics were found to be associated, which is relevant because the intervention consisted of both psychological content and self-monitoring. However, it must be noted that to draw conclusions on the effectiveness of the TK for weight loss, a more detailed analysis needs to be conducted by considering the potential confounders such as self-weighing frequency.

The eHealth literacy scores were compared with those reported in previous studies [25]. Overall, the score did not correlate with use and was not expected to affect TK use. The SUS scores measuring the usability of the TK ranged from 73 to 62.1 and were at their highest after 3 months of use. The scores were similar to the average SUS scores found in a large collection of studies and would correspond to a grade C, that is, satisfactory or average level of usability [26]. The *motivation and self-regulation* arm yielded the lowest usability scores. This may be because their intervention content comprised several interactive exercises compared with the *emotion regulation* arm that mainly comprised video and audio content and the control arm that comprised only the dashboard and graphs. Generally, user experience ratings decreased in all the arms during the 12-month follow-up period. The intervention arm participants gave significantly higher ratings to the TK regarding overall score, likelihood of recommending, intention to continue, and value (a TAMM dimension) compared with the control arm; otherwise, the control and intervention arm experiences were very similar. There were also very few differences among the intervention arms. In the TAMM dimensions, the perceived ease of use and perceived value ratings of the intervention arms were at a similar level as in the self-directed intervention in the study by Ma et al [27] and the decline during long-term use was of a similar magnitude. A more intensive coach-led intervention did not show similar declines in these dimensions [27].

The correlations between user experiences and use were relatively small, but they were consistently significant in the control and *emotion regulation* arms. There were some differences among the countries. The Portuguese participants in the intervention arms had significantly lower eHealth literacy scores than participants in other countries. However, interestingly, they were also the most positive in their user experiences, both in the control and intervention arms.

According to the qualitative findings, a major reason for deteriorated use and user experiences, were the technical difficulties, such as difficulties in logging in and the TK’s suboptimal performance on mobile devices that hindered its use. Although content and delivery could not be changed during the RCT, these findings highlight the need to reserve more time for iterative development and testing with target users and on different devices before starting a trial. The users were also disappointed by the lack of new and updated content in the TK and the absence of reminders after 18 weeks. It was decided early in the planning stages that there would be no updates to the TK or the content to avoid confounding of the results during the follow-up period. Furthermore, all the participants were given a Fitbit tracker and weight scales as part of the trial and although they were asked not to use the associated app, many of them used it and compared it with the TK, often unfavorably. These findings illustrate that, in the current digital health environment, even research-stage DBCIs need to be able to compete with commercial apps in their technical functioning and design. Resourcing of DBCI development and trial design need to allow for continuous content and design updates to avoid solutions becoming outdated before the end of the trial.

External factors also influenced the user experience and use of the TK. The users spoke about lifestyle-related barriers, such as being busy at work, going on a holiday, or having illnesses. These barriers have also been reported previously for mobile health apps [28]. DBCIs operate in challenging and uncontrolled environments and they should be able to anticipate and adapt to life events and have mechanisms to cope with them; for example, enable users to mute them for some time and then gently pull the user back to the service, especially in case of suspected relapse, as also suggested by Mattila et al [29].

Of the features, the reminder emails and self-monitoring–related tools (especially graphs, goal setting, and plans) were the most favorably experienced. Self-monitoring is one of the key behavior change techniques in weight management and is pervasive in both weight management treatments and commercial weight management apps [30,31]. Users are accustomed to and expect this feature; therefore, well-designed self-monitoring should be incorporated in DBCIs, while acknowledging that it may not work for everyone and that it may act through different mechanisms that are not yet fully understood [32]. The responsive features, especially the weight alerts, were also among the best-liked features.

Although generally enjoyed, there were mixed feelings regarding the intervention content, especially the emotion regulation content, which is a more novel approach. Although many participants appreciated it, some did not understand it, perhaps because it did not meet their expectations on what a WLM intervention should comprise. In contrast, some participants in the *motivation and self-regulation* arm expressed missing the emotion regulation aspect. These findings suggest that the approach and content of interventions should be personalized according to individual needs and preferences to ensure better acceptance.

Easier access through a smartphone app was most often mentioned as a technical improvement need. A social platform was also desired, as many participants had previous experiences with peer support helping in their weight management and hoped the TK would facilitate contact and engagement with peers. This feature was purposefully left out of the TK as it could have introduced uncontrolled or conflicting components to the intervention and biased the results.

Finally, the TK did not always match user expectations, which caused dissatisfaction and confusion. A clear introduction to the intervention approaches, requirements regarding devices and time allocation, and expected ways of use should be provided. Furthermore, as many participants received the trial procedures mixed with the TK, it is difficult to assess whether some of the negative experiences were related to the burden of trial procedures or the TK itself.

### Strengths and Limitations

The strengths of this study include a mixed methods design, large sample size, and focus groups conducted in different countries. In total, 85% (1383/1627) of the total study sample provided user experience responses at some point during the trial. However, we cannot ignore that the results represent the views of the more active users of the TK. Thus, it is not possible to predict the way in which this has skewed the findings. It is likely that active users used the TK more often because they had a positive experience. However, it is also possible that they were committed to using the TK regardless of their experience and were more exposed to the negative sides of the TK. Indeed, an association between negative user experience and more frequent use has been reported earlier by Tuch et al [14].

Similarly, focus groups are likely to include participants at the high end of activity and involvement with the intervention, which may also bias the findings. However, steps were taken to limit this bias. Participants were recruited using a ranking system, rather than using a direct convenience sample of participants who volunteered to participate.

### Future Work

Data-driven methods are expected to enable deeper personalization, adaptivity, and reactivity in DBCIs. For example, they enable the prediction, prevention, or detection of lapses in weight management behaviors [33,34]. Data from this trial may enable identification of specific profiles regarding weight maintenance and identification of the type of intervention content that is most effective or acceptable to the participants.

It is important to design DBCIs considering their intended way of use and make this clear to the user, that is, how often and for how long the intervention should be used. Here, the users were not always sure how they were supposed to use the TK after the active intervention. This study highlighted that continuous interaction with users is important and reminders are crucial in sustaining use. Reminder and interaction schemes should be designed to ensure effective engagement, but bearing in mind that making users dependent on an intervention is usually not a desirable goal [6].

Furthermore, the measurement of long-term user experience should be included as an integral part of DBCIs to guide further development, updates, and adaptation of the intervention. Similar to other aspects of an intervention, user experience varies between individuals and one solution is probably not optimal or pleasing for all.

### Conclusions

In the NoHoW RCT, most TK users were retained during the active intervention spanning the first 18 weeks of the study, when they were emailed weekly and reminded to access the intervention content. Technical difficulties related to access and use of the TK and the lack of new and updated content in TK hindered its use and user experiences. Personalized features, including the reminder emails, the self-monitoring–related tools, and weight alerts were well received and rated. These results highlight that the target users of DBCIs are already accustomed to using different types of existing health apps and services and are quick to compare new services with them. This emphasizes the need to finalize and evaluate the user experience and appearance of new services extensively before launching large-scale trials and to allow and enable updates of the content and design during the trial to avoid becoming outdated. Failure to provide an intervention with adequate application development, acceptance, and usability may undermine the original aims of the trial (in this case, testing the impact of self and emotion regulation strategies). Thus, DBCIs should be built to be more responsive and adaptive by monitoring the engagement, progress, and signs of relapse and reacting to those promptly to achieve continued sufficient level of engagement.

Future trials should focus not only on participants’ experience of mobile health devices but also on the full user experience of the interventions to ensure that the best possible support and experience is offered.

## Appendix

Multimedia Appendix 1 User experience questionnaire at month 1: first impressions questionnaire.

Multimedia Appendix 2 User experience questionnaires at months 3, 6, and 12. The user experience questionnaire was repeated at 3, 6, and 12 months.

Multimedia Appendix 3 Structured discussion guide.

Multimedia Appendix 4 Focus group code book.

Multimedia Appendix 5 Consort eHealth Checklist.

## Abbreviations

DBCI: digital behavior change intervention
eHEALS: eHealth Literacy Scale
RCT: randomized controlled trial
SUS: System Usability Scale
TAM: Technology Acceptance Model
TAMM: Technology Acceptance Model for Mobile Services
TK: Toolkit
UX1: user experience questionnaire at month 1
UX3: user experience questionnaire at month 3
UX6: user experience questionnaire at month 6
UX12: user experience questionnaire at month 12
WLM: weight loss maintenance
