# Supplementary material for: Users’ Experiences With the NoHoW Web-Based Toolkit With Weight and Activity Tracking in Weight Loss Maintenance: Long-term Randomized Controlled Trial
Source: J Med Internet Res. 2022 Jan 10;24(1):e29302. doi: 10.2196/29302 (PMC8787666; doi:10.2196/29302)
Supplement: Multimedia Appendix 1 [file jmir_v24i1e29302_app1.pdf]

English

Default Question Block

First Impressions of the NoHoW Toolkit

The following questions measure your first impressions on the NoHoW Toolkit. Please respond to the following statements with the option that best describes your experience.

	Strongly disagree 1	2	3	4	Strongly Agree 5
It was easy to start using the Toolkit.	○	○	○	○	○
The Toolkit is intuitive to use.	○	○	○	○	○
I did not need a lot of instructions to start using the Toolkit.	○	○	○	○	○
It is easy to find what I need in the Toolkit.	○	○	○	○	○
The Toolkit is easy to use.	○	○	○	○	○
It is easy to navigate in the Toolkit.	○	○	○	○	○
I find the Toolkit visually pleasing.	○	○	○	○	○
I find the Toolkit to					

be interesting.	○	○	○	○	○
I believe the Toolkit can help me in weight management.	○	○	○	○	○
I enjoy using the Toolkit.	○	○	○	○	○
I trust the information the Toolkit provides me.	○	○	○	○	○
I trust the Toolkit keeps my personal data safe.	○	○	○	○	○
The Toolkit works reliably.	○	○	○	○	○
I have not encountered any technical problems when using the Toolkit.	○	○	○	○	○

What overall score would you give to the service based on your experience so far? (Where 0 is very bad and 10 is very good).

1	2	3	4	5	6	7	8	9	10
○	○	○	○	○	○	○	○	○	○

How likely is it that you would consider using the service in the future?

Not at all likely 0	1	2	3	4	5	6	7	8	9	Extremely Likely 10
○	○	○	○	○	○	○	○	○	○	○

How likely is it that you would recommend the service to a friend or colleague?

Not at all likely 0	1	2	3	4	5	6	7	8	9	Extemely Likely 10
○	○	○	○	○	○	○	○	○	○	○

If you have any other feedback on the Toolkit, you may write it here:

Please notice that if you want to be contacted by the research team e.g. in the case of unsolved problems, you may contact us through the Toolkit. Thank you for your responses!

Powered by Qualtrics
