# Supplementary material for: Users’ Experiences With the NoHoW Web-Based Toolkit With Weight and Activity Tracking in Weight Loss Maintenance: Long-term Randomized Controlled Trial
Source: J Med Internet Res. 2022 Jan 10;24(1):e29302. doi: 10.2196/29302 (PMC8787666; doi:10.2196/29302)
Supplement: Multimedia Appendix 2 [file jmir_v24i1e29302_app2.pdf]

English

## Default Question Block

User experience questionnaire

The following questions measure your experiences on using the NoHoW Toolkit. Please respond to the following statements with the option that best describes your experience.

On average, how frequently did you use the Toolkit during the study?

- Very Frequently
- Frequently
- Occasionally
- Rarely
- Very Rarely
- Never

What option describes your Toolkit use behavior best?

- 1. I used the Toolkit very constantly during the whole study
- 2. I used the Toolkit more in the beginning of the study
- 3. I used the Toolkit more in the end of the study
- 4. I quit using the Toolkit in the middle of the study
- ______ 5. Other

What motivated you to continue using the Toolkit?

Why did you use the Toolkit more in the beginning than in the end of the study?

Why did you start to use the Toolkit in the end of the study?

Why did you quit using the Toolkit in the middle of the study?

Please respond to the following statements concerning the overall user experience of the NoHow Toolkit.

| | Strongly Disagree 1 | 2 | 3 | 4 | Strongly Agree 5 |
|---|---|---|---|---|---|
| It was easy to learn to use the | ○ | ○ | ○ | ○ | ○ |

| | | | | | |
|---|---|---|---|---|---|
| Toolkit. | | | | | |
| It was easy to fit using the Toolkit into my life. | ○ | ○ | ○ | ○ | ○ |
| It is easy to find what I need in the Toolkit. | ○ | ○ | ○ | ○ | ○ |
| The Toolkit guides me to useful information. | ○ | ○ | ○ | ○ | ○ |
| I enjoy using the Toolkit. | ○ | ○ | ○ | ○ | ○ |
| I believe the Toolkit can help me manage my weight. | ○ | ○ | ○ | ○ | ○ |
| I found the Toolkit visually pleasing. | ○ | ○ | ○ | ○ | ○ |
| The information provided by the Toolkit is useful to me. | ○ | ○ | ○ | ○ | ○ |
| The Toolkit does everything I would expect. | ○ | ○ | ○ | ○ | ○ |
| I like receiving email from the Toolkit. | ○ | ○ | ○ | ○ | ○ |
| I would like to continue using the Toolkit. | ○ | ○ | ○ | ○ | ○ |
| I trust the Toolkit keeps my personal data safe. | ○ | ○ | ○ | ○ | ○ |
| I can trust the information and | ○ | ○ | ○ | ○ | ○ |

| | | | | | |
|---|---|---|---|---|---|
| advice I get from the Toolkit. | | | | | |
| The Toolkit works reliably. | ○ | ○ | ○ | ○ | ○ |
| I did not encounter any technical problems when using the Toolkit. | ○ | ○ | ○ | ○ | ○ |

Please respond to the following statements concerning the general usability of the NoHow Toolkit.

| | Strongly Disagree 1 | 2 | 3 | 4 | Strongly Agree 5 |
|---|---|---|---|---|---|
| I think that I would like to use this system frequently. | ○ | ○ | ○ | ○ | ○ |
| I found the system unnecessarily complex. | ○ | ○ | ○ | ○ | ○ |
| I thought the system was easy to use. | ○ | ○ | ○ | ○ | ○ |
| I think that I would need the support of a technical person to be able to use this system. | ○ | ○ | ○ | ○ | ○ |
| I found the various functions in this system were well integrated. | ○ | ○ | ○ | ○ | ○ |
| I thought there was too much inconsistency in this system. | ○ | ○ | ○ | ○ | ○ |

| | | | | | |
|---|---|---|---|---|---|
| I would imagine that most people would learn to use this system very quickly. | ○ | ○ | ○ | ○ | ○ |
| I found the system very cumbersome to use. | ○ | ○ | ○ | ○ | ○ |
| I felt very confident using the system. | ○ | ○ | ○ | ○ | ○ |
| I needed to learn a lot of things before I could get going with this system. | ○ | ○ | ○ | ○ | ○ |

Please rate HOW IMPORTANT the following features are to you:

| | Not at all important 1 | 2 | 3 | 4 | Very important 5 | Can't say/ unfamiliar with the feature |
|---|---|---|---|---|---|---|
| Interactive map | ○ | ○ | ○ | ○ | ○ | ○ |
| Theme introduction videos | ○ | ○ | ○ | ○ | ○ | ○ |
| Textual information provided in the sessions | ○ | ○ | ○ | ○ | ○ | ○ |
| Interactive exercises in the sessions (e.g. weight history graph, quizzes) | ○ | ○ | ○ | ○ | ○ | ○ |
| Audio exercises | | | | | | |

| | | | | | | |
|---|---|---|---|---|---|---|
| in the sessions (e.g. mindful eating exercise) | ○ | ○ | ○ | ○ | ○ | ○ |
| Dashboard (i.e. home view) | ○ | ○ | ○ | ○ | ○ | ○ |
| Graphs (e.g. weight, ratings of diet & mood) | ○ | ○ | ○ | ○ | ○ | ○ |
| Goal setting | ○ | ○ | ○ | ○ | ○ | ○ |
| Coping and action plan | ○ | ○ | ○ | ○ | ○ | ○ |
| Personal notes | ○ | ○ | ○ | ○ | ○ | ○ |
| Personal feedback tile | ○ | ○ | ○ | ○ | ○ | ○ |
| Weight alert | ○ | ○ | ○ | ○ | ○ | ○ |
| Summary tile | ○ | ○ | ○ | ○ | ○ | ○ |
| Weekly emails | ○ | ○ | ○ | ○ | ○ | ○ |

Please rate HOW EASY TO USE the following features are to you:

| | Not at all important 1 | 2 | 3 | 4 | Very important 5 | Can't say/ unfamiliar with the feature |
|---|---|---|---|---|---|---|
| Interactive map | ○ | ○ | ○ | ○ | ○ | ○ |
| Theme introduction videos | ○ | ○ | ○ | ○ | ○ | ○ |
| Textual information provided in the sessions | ○ | ○ | ○ | ○ | ○ | ○ |

| | | | | | | |
|---|---|---|---|---|---|---|
| Interactive exercises in the sessions (e.g. weight history graph, quizzes) | ○ | ○ | ○ | ○ | ○ | ○ |
| Audio exercises in the sessions (e.g. mindful eating exercise) | ○ | ○ | ○ | ○ | ○ | ○ |
| Dashboard (i.e. home view) | ○ | ○ | ○ | ○ | ○ | ○ |
| Graphs (e.g. weight, ratings of diet & mood) | ○ | ○ | ○ | ○ | ○ | ○ |
| Goal setting | ○ | ○ | ○ | ○ | ○ | ○ |
| Coping and action plan | ○ | ○ | ○ | ○ | ○ | ○ |
| Personal notes | ○ | ○ | ○ | ○ | ○ | ○ |
| Personal feedback tile | ○ | ○ | ○ | ○ | ○ | ○ |
| Weight alert | ○ | ○ | ○ | ○ | ○ | ○ |
| Summary tile | ○ | ○ | ○ | ○ | ○ | ○ |
| Weekly emails | ○ | ○ | ○ | ○ | ○ | ○ |

Please rate HOW CONVENIENT the different features of the Toolkit are to you:

| | Not at all important 1 | 2 | 3 | 4 | Very important 5 | Can't say/ unfamiliar with the feature |
|---|---|---|---|---|---|---|
| Interactive map | ○ | ○ | ○ | ○ | ○ | ○ |

| | | | | | | |
|---|---|---|---|---|---|---|
| Theme introduction videos | ○ | ○ | ○ | ○ | ○ | ○ |
| Textual information provided in the sessions | ○ | ○ | ○ | ○ | ○ | ○ |
| Interactive exercises in the sessions (e.g. weight history graph, quizzes) | ○ | ○ | ○ | ○ | ○ | ○ |
| Audio exercises in the sessions (e.g. mindful eating exercise) | ○ | ○ | ○ | ○ | ○ | ○ |
| Dashboard (i.e. home view) | ○ | ○ | ○ | ○ | ○ | ○ |
| Graphs (e.g. weight, ratings of diet & mood) | ○ | ○ | ○ | ○ | ○ | ○ |
| Goal setting | ○ | ○ | ○ | ○ | ○ | ○ |
| Coping and action plan | ○ | ○ | ○ | ○ | ○ | ○ |
| Personal notes | ○ | ○ | ○ | ○ | ○ | ○ |
| Personal feedback tile | ○ | ○ | ○ | ○ | ○ | ○ |
| Weight alert | ○ | ○ | ○ | ○ | ○ | ○ |
| Summary tile | ○ | ○ | ○ | ○ | ○ | ○ |
| Weekly emails | ○ | ○ | ○ | ○ | ○ | ○ |

Please rate HOW ENJOYABLE you have found using the following features:

Can't

| | Not at all important 1 | 2 | 3 | 4 | Very important 5 | say/ unfamiliar with the feature |
|---|---|---|---|---|---|---|
| Interactive map | ○ | ○ | ○ | ○ | ○ | ○ |
| Theme introduction videos | ○ | ○ | ○ | ○ | ○ | ○ |
| Textual information provided in the sessions | ○ | ○ | ○ | ○ | ○ | ○ |
| Interactive exercises in the sessions (e.g. weight history graph, quizzes) | ○ | ○ | ○ | ○ | ○ | ○ |
| Audio exercises in the sessions (e.g. mindful eating exercise) | ○ | ○ | ○ | ○ | ○ | ○ |
| Dashboard (i.e. home view) | ○ | ○ | ○ | ○ | ○ | ○ |
| Graphs (e.g. weight, ratings of diet & mood) | ○ | ○ | ○ | ○ | ○ | ○ |
| Goal setting | ○ | ○ | ○ | ○ | ○ | ○ |
| Coping and action plan | ○ | ○ | ○ | ○ | ○ | ○ |
| Personal notes | ○ | ○ | ○ | ○ | ○ | ○ |
| Personal feedback tile | ○ | ○ | ○ | ○ | ○ | ○ |
| Weight alert | ○ | ○ | ○ | ○ | ○ | ○ |
| Summary tile | ○ | ○ | ○ | ○ | ○ | ○ |
| Weekly emails | ○ | ○ | ○ | ○ | ○ | ○ |

Please rate HOW SATISFIED you have been with the following features:

| | Not at all important 1 | 2 | 3 | 4 | Very important 5 | Can't say/ unfamiliar with the feature |
|---|---|---|---|---|---|---|
| Interactive map | ○ | ○ | ○ | ○ | ○ | ○ |
| Theme introduction videos | ○ | ○ | ○ | ○ | ○ | ○ |
| Textual information provided in the sessions | ○ | ○ | ○ | ○ | ○ | ○ |
| Interactive exercises in the sessions (e.g. weight history graph, quizzes) | ○ | ○ | ○ | ○ | ○ | ○ |
| Audio exercises in the sessions (e.g. mindful eating exercise) | ○ | ○ | ○ | ○ | ○ | ○ |
| Dashboard (i.e. home view) | ○ | ○ | ○ | ○ | ○ | ○ |
| Graphs (e.g. weight, ratings of diet & mood) | ○ | ○ | ○ | ○ | ○ | ○ |
| Goal setting | ○ | ○ | ○ | ○ | ○ | ○ |
| Coping and action plan | ○ | ○ | ○ | ○ | ○ | ○ |
| Personal notes | ○ | ○ | ○ | ○ | ○ | ○ |
| Personal | | | | | | |

| | | | | | | |
|---|---|---|---|---|---|---|
| feedback tile | ○ | ○ | ○ | ○ | ○ | ○ |
| Weight alert | ○ | ○ | ○ | ○ | ○ | ○ |
| Summary tile | ○ | ○ | ○ | ○ | ○ | ○ |
| Weekly emails | ○ | ○ | ○ | ○ | ○ | ○ |

Please rate HOW MOTIVATED you are to continue using the following features:

| | Not at all important 1 | 2 | 3 | 4 | Very important 5 | Can't say/ unfamiliar with the feature |
|---|---|---|---|---|---|---|
| Interactive map | ○ | ○ | ○ | ○ | ○ | ○ |
| Theme introduction videos | ○ | ○ | ○ | ○ | ○ | ○ |
| Textual information provided in the sessions | ○ | ○ | ○ | ○ | ○ | ○ |
| Interactive exercises in the sessions (e.g. weight history graph, quizzes) | ○ | ○ | ○ | ○ | ○ | ○ |
| Audio exercises in the sessions (e.g. mindful eating exercise) | ○ | ○ | ○ | ○ | ○ | ○ |
| Dashboard (i.e. home view) | ○ | ○ | ○ | ○ | ○ | ○ |
| Graphs (e.g. weight, ratings of diet & mood) | ○ | ○ | ○ | ○ | ○ | ○ |

| | | | | | | |
|---|---|---|---|---|---|---|
| Goal setting | ○ | ○ | ○ | ○ | ○ | ○ |
| Coping and action plan | ○ | ○ | ○ | ○ | ○ | ○ |
| Personal notes | ○ | ○ | ○ | ○ | ○ | ○ |
| Personal feedback tile | ○ | ○ | ○ | ○ | ○ | ○ |
| Weight alert | ○ | ○ | ○ | ○ | ○ | ○ |
| Summary tile | ○ | ○ | ○ | ○ | ○ | ○ |
| Weekly emails | ○ | ○ | ○ | ○ | ○ | ○ |

What overall score would you give to the service?

| 0 | 1 | 2 | 3 | 4 | 5 | 6 | 7 | 8 | 9 | 10 |
|---|---|---|---|---|---|---|---|---|---|---|
| ○ | ○ | ○ | ○ | ○ | ○ | ○ | ○ | ○ | ○ | ○ |

How likely is it that you would consider using the service in the future?

| Not at all likely 0 | 1 | 2 | 3 | 4 | 5 | 6 | 7 | 8 | 9 | Extremely Likely 10 |
|---|---|---|---|---|---|---|---|---|---|---|
| ○ | ○ | ○ | ○ | ○ | ○ | ○ | ○ | ○ | ○ | ○ |

How likely is it that you would recommend the service to a friend or colleague?

| Not at all likely 0 | 1 | 2 | 3 | 4 | 5 | 6 | 7 | 8 | 9 | Extremely Likely 10 |
|---|---|---|---|---|---|---|---|---|---|---|
| ○ | ○ | ○ | ○ | ○ | ○ | ○ | ○ | ○ | ○ | ○ |

If you would like to give additional feedback on the Toolkit, please write it here:

Thank you for your valuable responses!

Powered by Qualtrics
