# Supplementary material for: Users’ Experiences With the NoHoW Web-Based Toolkit With Weight and Activity Tracking in Weight Loss Maintenance: Long-term Randomized Controlled Trial
Source: J Med Internet Res. 2022 Jan 10;24(1):e29302. doi: 10.2196/29302 (PMC8787666; doi:10.2196/29302)
Supplement: Multimedia Appendix 3 [file jmir_v24i1e29302_app3.pdf]

Questions – Focus group

Ask the main question under each theme and use probes when needed (i.e. if the participants do not bring up the topics in the probes). Please make sure probes in ***bolded italics*** are discussed. Try to encourage participants to interact with each other as focus group is a group process.

A fairly standard format is to involve around six to eight people in one focus group. The suggested time for the focus group is an hour-and-a-half to two hours.

1. Introduction

- Introductions.
- Goals: The purpose of this focus group is to collect your experiences on using the Toolkit. The information you provide will be used to gain deeper knowledge on the convenience and ease of use of the Toolkit as well as outcomes and unintended consequences of its use. We appreciate honesty and welcome any criticism you may have.
- This focus group will take about one and a half hours and we will cover several themes, ranging from your initial expectations to discussing specific features of the Toolkit.

2. Theme: Expectations and needs

What did you expect from the Toolkit when you joined the study?

Probes:

- ***Did you have a need for this type of service when you joined the study?***
- Do you feel that web-based services could help you in weight management?
- What did you expect from the Toolkit?

3. Theme: Ease of adoption and ease of use

What did you think about the ease of use of the Toolkit?

Probes:

- ***Was it easy to take the Toolkit into use and learn to use it?***
- Do you feel that you had the skills and know-how needed to use this Toolkit? Would you have liked to have more guidance?
- ***Was the Toolkit generally easy to use and navigate?***
- Was it easy to find information in the Toolkit?

4. Theme: Visual appearance of the Toolkit

What did you think about the visual appearance of the Toolkit?

Probes:

- ***Were there things you especially liked or disliked about the visual appearance of the Toolkit?***
- Did you find the Toolkit attractive?
- Was the look and feel of the Toolkit competent/professional/trustworthy?

5. Theme: Content of the Toolkit

What did you think about the information provided by the Toolkit?

Probes:

- ***Did the information feel new to you? Can you give an example of something that was very new to you? Did you already know the information in the Toolkit?***
- ***Did you like the ways in which the information was presented, e.g. videos, audio, interactive exercises, text, images?***
- What did you think about the theme introduction videos?
- What did you think about the textual information provided in the sessions?
- What did you think about the interactive exercises in the sessions?
- What did you think about the audio exercises in the sessions?
- ***Do you feel the information provided in the Toolkit could help you in weight management? Why or why not?***
- Did you expect different kind of information or different ways of presenting the information?

6. Theme: Other functionalities of the Toolkit

What did you feel about the other functionalities of the Toolkit?

Probes:

- ***How did you feel about the interactive map of the Toolkit?***
- Was it easy to find the right session in the project map?
- ***How did you feel about the dashboard (i.e. home view) with the tiles?***
- Was the dashboard useful for you? In what situations did you use it?
- ***What did you feel about the graphs (i.e. weight, ratings of diet and mood)?***
- ***What did you think about the goal setting and coping and action plans in the weight tile?***
- ***What did you think about the personal notes?***
- Did you use that functionality?
- Was it useful for you? How?
- **What did you think about the personal feedback tile?**
- Did you receive any feedback in the personal feedback tile?
- Was it useful for you? How?
- **What did you think about the weight alert?**
- Did you receive weight alert? If you do not feel comfortable discussing about it, you don't have to.
- Was it useful for you? How?
- ***What did you feel about the summary tile?***
- Did you use that functionality?
- Was it useful for you? How?
- ***What did you think about the email prompts you received from the Toolkit?***
- Was the frequency of the email prompts suitable for you?
- Was the content of the emails interesting?

7. Theme: Ways of use

Can you describe how you used the Toolkit during the study?

Probes:

- ***What device did you use to access the Toolkit? Did it work well on that device? Why or why not?***
- ***How much time did it take to use the Toolkit at each visit? Was it acceptable?***

- ***What made you visit the Toolkit? (e.g. email prompts or some other reason)***
- Did you find it difficult to remember to use the Toolkit?
- ***Where did you use the Toolkit (e.g. home, work, commuting)?***
- Did you feel some environments were better suited for using the Toolkit than others?
- Did you use the Toolkit with someone else or show it to someone?
- ***Was there something that distracted you from using the Toolkit?***
- ***Was it easy to use the Toolkit together with the Fitbit monitoring devices?***
- Did you face any problems?
- Did the Toolkit bring additional value over Fitbit health monitoring?

8. Theme: Impact

What was the main outcome for you from the study?

Probes:

- ***Did you learn something useful by using the Toolkit? What?***
- ***Did using the Toolkit change your weight management related thinking or behaviours? How?***
- ***Were there some unintended consequences?***
- ***Did the Toolkit use have any impact on you social interactions with other people?***
- Did you show the Toolkit for other people? How they responded?
- How your weight management efforts influenced on your social relationships?

9. Conclusions

What did you take away from the study?

Probes:

- Do you feel that the Toolkit could be useful to you in weight management in the future?
- Do you have ideas or suggestions for improvements?

Thank you!
