# Supplementary material for: Users’ Experiences With the NoHoW Web-Based Toolkit With Weight and Activity Tracking in Weight Loss Maintenance: Long-term Randomized Controlled Trial
Source: J Med Internet Res. 2022 Jan 10;24(1):e29302. doi: 10.2196/29302 (PMC8787666; doi:10.2196/29302)
Supplement: Multimedia Appendix 4 [file jmir_v24i1e29302_app4.pdf]

NoHoW

Nodes

Name	Description
Around the NoHoW toolkit	Other subjects not directly concerning the NoHoW toolkit, but still of interest.
Confusing the Toolkit with trial-related tasks	
Support elsewhere - past and present	This includes both current and earlier support
Local slimming groups	
Other activity trackers (e.g. Polar, Garmin)	
Other apps (e.g. Lifesum, Endomondo)	
Other ways of getting support for weight loss or maintenance	Some Danish participants have attended the same "højskole"
Slimming World	
Wrong questionnaire	

Name	Description
Improvement suggestions	Some mention that the graphs could be more useful, if one could combine them and make it easier to see the correlations between them.
Create a NoHoW app	
Diary of personal insights	
Feedback based on data	
Food tracker	
List of weights	
Reboot option to start over	
Support groups	
Warn about expected time consumption on NoHoW website	Some participants, who have been exposed to the rather long meditation videos, have had to interrupt in the middle of it, because they were not warned in advanced that it would take that much time.
New and interesting information	
Usefulness	In this category, opinions about the feature of both the NoHoW website and the Fitbit app are collected.
About specific tools on NoHoW website	

Name	Description
Audio	
Dashboard	
Diary	
Graphs	
Map	About the map in top of the dashboard
Quizzes	
Sessions	
Testimonies	
Videos	About the usefulness of the videos on the NoHoW website.
Weight alert	
Benefits	This includes the benfits of using the NoHoW toolkit, including Fitbit.
Enables to act on data	
Enjoy using the Toolkit	
Enjoyed mindfulness exercises	
Enjoyed using Fitbit	

Name	Description
Helpful and supportive	
Inducing reflections about mood, eating, weight etc.	
Keeps you on track and focused	
Helps get back on track	
Motivating	
Thought-provoking	
Visualization of development and progress	Opinions about the graphs/visualization of development and progress
Comparing to commercial services	
Comparing NoHoW website and Fitbit app	Some compare the website with the app. It is for instance common that people think that they do not need NoHoW, because Fitbit has everything they need (and that they would have used the NoHoW website more, was it not for Fitbit).
Information on NoHoW website	About the information offered by the NoHoW website
Negative	
Condescending	Some describe the content or parts of the content as condescending or "American self-help".

Name	Description
More information and advice desired	
Not scientific enough	
Old news - 'I knew it all'	Some express that the information provided on the NoHoW website has been old news to them, becaue they are experiences weight losers.
Posh or 'academic' language	
Uncertain whether to trust the information - 'It's not up to date'	Some do not have much faith in scientists, who keep changing their mind.
Positive	
Monitoring	About everything that is monitored, that you do not have to register yourself. That is: everything that comes through the Fitbit. Also bout everything that is registered, that is: that you have to plot in yourself.
Links between variables	
Negative	
Positive	
Not useful	A gathering of expressions, where people tell how and why the tools (or some of the tools), they were offered, were not useful to them.

Name	Description
Disappointed & failed expectations	
Food-related tools missing	
Not compatible with personal needs and preferences	
Reminders and other automatically generated messages from NoHoW	
E-mail reminder from NoHoW	
More reminders desired	
Reminder on calendar or phone	
Weekly emails as the motivation to continue	
User experience	
Barriers	
Boring and repetitive	
Busy life - life takes over	

Name	Description
Confused about the content and usage	
Not enough instructions	
Setting goals	
Does not like the 'mindfulness' part	
Forgetting to use the Toolkit	
Hard to access	
Having to log in every time & find the password from emails	
Having to use a computer	
Not optimized for all devices & browsers	
Not a fan of technology	
Time-consuming & need to find suitable place	
Too complicated	

Name	Description
Too simple	
Using NoHoW web site because of sense of obligation	
Design	People express different opinions about design.
Navigation	
Pacing	
Progress indicators for session completion and status in program	
Too large font - other people can see	
User interface	
Frequency	
Frequent or regular	
Infrequent or irregular	
New then same old same old	

Name	Description
Not knowing the intended frequency	
Binging on sessions	
Self-directed	
When getting a reminder	
Place of use	Where the NoHoW platform has been used.
Home	
Work	
Technical issues	
Fitbit	
Does not support older operating systems	
Fitbit wristband causing eczema etc	Some have experiences some physically uncomfortable reactions
Misregistration of activities - precision wanted	Fitbit registers some activities as others or do not register at all, or registers too much! They participants want precision!

Name	Description
Scale issues	Some mention that it is not accurate or that it doesn't communicate well with the website
NoHoW website	
Does not save information	It is mentioned that sometimes at the NoHoW website, you fill in information (such as goal setting), but then it doesn't save it, and you can't re-find it.
Fitbit sync issues	
Other technical difficulties	
Problems in setting goals	
Sessions marked as done when just browsing	
Website login issues	Some complain about login issues; that you cannot change your password or that it doesn't remember your password. This stops some from actuallly using the website.
Website star system	Some express technical difficulties, when attributing stars on the NoHoW website.
Wrong weight unit	
User-friendliness	
Not user-friendly	
User-friendly	

Nodes\\UX_12MO_CPH

Name	Description
feedback_on_toolkit	
motivation_to_continue	
why_quit_using	
why_used_more_at_beginning	

Nodes\\UX_12MO_LIS

Name	Description
feedback_on_toolkit	
motivation_to_continue	
why_quit_using	
why_used_more_at_beginning	

Nodes\\UX_12MO_UL

Name	Description
feedback_on_toolkit	
motivation_to_continue	
why_quit_using	
why_used_more_at_beginning	

Nodes\\UX_3MO_CPH

Name	Description
feedback_on_toolkit	
motivation_to_continue	
why_quit_using	
why_used_more_at_beginning	

Nodes\\UX_3MO_LIS

Name	Description
feedback_on_toolkit	
motivation_to_continue	
why_quit_using	
why_used_more_at_beginning	

Nodes\\UX_3MO_UL

Name	Description
Column 3	
Column 4	
Column 5	
feedback_on_toolkit	
motivation_to_continue	
why_quit_using	

Name	Description
why_used_more_at_beginning	

Nodes\\UX_3MO_UL (2)

Name	Description
Column 3	
Column 4	
Column 5	
Column 6	

Nodes\\UX_6MO_CPH

Name	Description
feedback_on_toolkit	
motivation_to_continue	
why_quit_using	
why_used_more_at_beginning	

Nodes\\UX_6MO_LIS

Name	Description
feedback_on_toolkit	
motivation_to_continue	
why_quit_using	
why_used_more_at_beginning	

Nodes\\UX_6MO_UL

Name	Description
feedback_on_toolkit	
motivation_to_continue	
why_quit_using	
why_used_more_at_beginning	

Nodes\\UX_FI_CPH

Name	Description
feedback_on_toolkit	

Nodes\\UX_FI_LIS

Name	Description
feedback_on_toolkit	

Nodes\\UX_FI_UL

Name	Description
Column 3	
feedback_on_toolkit	
